# Combining PC-SAFT and ML to Access Unknown API Solubilities

**DOI:** 10.1021/acs.molpharmaceut.5c01889

**Published:** 2026-04-20

**Authors:** Jonas Habicht, Gabriele Sadowski, Christoph Brandenbusch

**Affiliations:** † 14311 TU Dortmund University, Laboratory of Thermodynamics, Department of Biochemical and Chemical Engineering, Emil-Figge-Str. 70, Dortmund 44227, Germany; ‡ Amofor GmbH, Otto-Hahn-Strasse 15, Dortmund 44227, Germany

**Keywords:** PC-SAFT, machine learning, neural networks, solubility, API

## Abstract

Predicting the solubility of active pharmaceutical ingredients (APIs) is essential throughout drug development. However, state-of-the-art modeling approaches require system-specific data sets for parameter estimation and are resource intensive. This work introduces a new method that integrates adaptive machine learning (ML) methods with PC-SAFT modeling to vastly reduce requirements of experimental data. Instead of extensive experimental campaigns of solubility measurements, only the molecular structure and melting properties of the API are needed - information onten available in literature or easily measured. The ML framework applied in this work supplies PC-SAFT parameters for APIs. With solvent parameters already available from literature, this novel approach provided highly accurate solubility estimations for 21 APIs in pure solvents (*R* ^2^ = 0.83 without using any binary data and *R* ^2^ = 0.98 using a single binary data point), as well as for mixed solvents, closely matching literature data. Compared to prior models, this hybrid method is more generalizable, consistent, and efficient, streamlining the workflow and providing reliable predictions with minimal experimental effort. By making a thermodynamic-based solubility assessment available early in process development, it outperforms state-of-the-art models that demand significantly more experimental input. The results of this work indicate that the newly developed ML framework can be efficiently applied to provide PC-SAFT parameters for APIs with minimal need of or even without using any experimental solubility data, which can be used to achieve thermodynamics-based access to API solubility in a very early stage of process development. This approach does not only provide solubility data in pure solvents but also in solvent mixtures.

## Graphical abstract

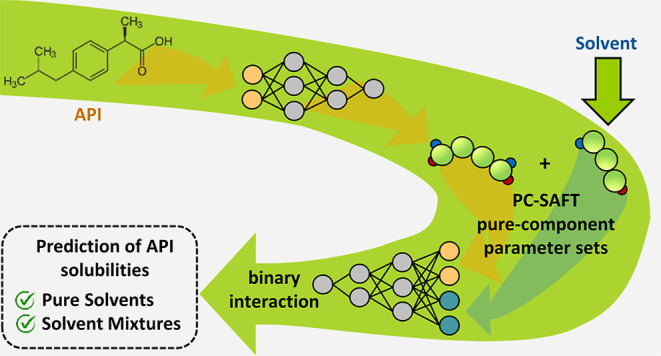

## 1 Introduction

The solubility of an active pharmaceutical ingredient (API) is a key physical property, required for formulation development, designing manufacturing processes, and performing bioavailability assessments.^1^ Knowledge of solubility further enables the prediction of (complex) phase behaviors essential to drug development, such as recrystallization risks of the API, or the phase separation propensity in amorphous solid dispersions^2^ at storage conditions. A modeling-based access to API solubility in pure or mixed solvents can assist industry standard wet methods, leading to more efficient product and process development.^3^ Thermodynamic modeling in particular, has emerged as reliable addition in formulation optimization and process development,^4,5^ with advanced physically sound approaches, such as PC-SAFT (Perturbed-Chain Statistical Associating Fluid Theory^6^), showing broad applicability in various pharmaceutical applications.^7–10^

Different approaches for API solubility predictions, usually focusing on particular solvents only, are already available in the literature. Quantitative structure–property relations^4^ can predict the aqueous solubility of organic molecules (e.g., acetic acid, tetrahydrofuran) and APIs (e.g., nifedipine, diazepam) but are limited to pure water as the solvent. Most classical ML-approaches (using different algorithms such as random forests or neural networks)^5–7^ also allow for solubility predictions, but are mostly limited to a specific solvent or solvent systems and thus lack flexibility regarding arbitrary solvents. Circumventing this drawback, Cenci et al.^6^ developed a partial least-squares regression method to train a solubility model using UNIFAC subgroups as input, thus not being limited to specific solvents or solvent mixtures. However, this model lacks transferability to novel molecules, as the UNIFAC groups need to be available. Furthermore, all of the approaches mentioned above only allow for solubility predictions. If other properties, such as liquid density or liquid–liquid phase separation, are of interest, different models have to be used. This can lead to inconsistent data sets because calculation results are based on multiple models that require different inputs. For instance, one model may require the chemical structure as input, while another may need specific groups, UNIFAC groups, ECFP fingerprints, density, or vapor pressure data, resulting in outputs that cannot be directly compared.

Moreover, ML models that aim to calculate solubility data directly face several inherent issues. The main challenge of these approaches is not just the availability of experimental input data. The even bigger problem is the—often unknown—reliability of experimental solubility data used for training purposes. Many APIs form different polymorphs. Therefore, an API-solubility measurement does not only require analyzing the saturated liquid but also requires an analysis (e.g., via PXRD or DSC) of the solid that is in equilibrium with that liquid (i.e., at the end of the experiment) to know the solubility of which polymorph has actually been measured. The same applies to the formation of hydrates or solvates which also might form during a solubility measurement and of course have a different solubility than the pure API. For aqueous solubility data, the situation is even worse as, in addition to the solid-state problem, quite often those measurements are performed in the presence of buffers, pH-changing agents or electrolytes, which of course have a considerable impact on API solubility.^8^ Thus, evaluating and carefully selecting the solubility data used as a training set is the main challenge for any ML approach.

In contrast, thermodynamic models such as PC-SAFT do allow for a consistent and all-encompassing access not only to solubility data but to any kind of phase equilibria and physical properties of the API-solvent system, such as solubility of the crystalline or amorphous compounds, liquid–liquid miscibility or even vapor-sorption data as a function of relative humidity or solvent partial pressure.^9,10^ On the downside, these thermodynamic models require some effort and expertise to determine the model parameters necessary for conducting those calculations. This is especially true in the case of APIs, as experimental data typically used for parametrization/fitting of PC-SAFT pure-component parameters (that is vapor pressure and saturated-liquid densities) are not available for those components. To overcome this drawback, we recently developed a two-level Machine Learning (ML) approach,^11^ enabling rapid and accurate predictions of pure-component parameter sets and even binary interaction parameters solely based on the molecular structure of a given target molecule and/or binary mixture.^12^ With this new approach, traditional modeling can be accelerated by requiring less experimental data (at minimum the melting properties of the API) and thus guiding present-day wet measurements more efficiently.

Within this work, our previously developed two-level ML approach^11^ is used to (1) access PC-SAFT pure-component parameters of APIs and (2) to obtain binary interaction parameters between solvent and API. This procedure ensures reliability as ML-derived parameters are used alongside established PC-SAFT solvent parameter sets. Solubilities were calculated in this work for a total of 21 APIs in 27 solvents. Additionally, the solubility of 4 APIs in 7 solvent mixtures was considered. If experimental data were used from literature sources, it was ensured that an analysis of the equilibrium solid had been performed to account for different polymorphic forms of the same API.

In contrast to available models, this hybrid approach offers a flexible solubility calculation based on both ML-derived PC-SAFT parameters (API) and established PC-SAFT parameters (solvents). This strategy streamlines the traditionally complex and resource-intensive modeling workflow. As a result, researchers and formulators gain access to reliable, consistent, and flexible solubility calculations across a wide range of solvent systems.

## 2 Computational Methods

### 2.1 Solubility Calculation with PC-SAFT

The calculation of the solubility *x*_API_^L^ at a temperature *T* is performed using a thermodynamic approach according to eq 1 requiring the activity coefficient γ_API_^L^of the API and the three melting properties namely the melting temperature *T*_API_^SL^, the melting enthalpy Δ*h*_API_^SL^, and the difference of heat capacity between solid and liquid state of the API Δ*c*_p_^SL^.

1 $$x_{API}^{L}=\frac{1}{\gamma_{API}^{L}}\exp\left[-\frac{{\Delta h}_{API}^{SL}}{RT}\left(\frac{T_{API}^{SL}-T}{T_{API}^{SL}}\right)-\frac{{\Delta c}_{p}^{SL}}{R}\left(\ln\left(\frac{T_{API}^{SL}}{T}\right)+\frac{T-T_{API}^{SL}}{T}\right)\right]$$

the solubility is calculated as mole fraction of the API in the liquid phase in this work, which is related to the solubility *S* measured in g·L^–1^ through the molar masses of the API *M*_API_ and the solvent *M*_solvent_, and the solvent density ρ_solvent_ as shown in eq 2.

2 $$S\left[\frac{g}{L}\right]=\rho_{solvent}\left[\frac{g}{L}\right]\cdot \frac{x_{API}^{L}}{1-x_{API}^{L}}\cdot \frac{M_{solvent}}{M_{API}}$$

The activity coefficient of the API is calculated with PC-SAFT from the pure-component parameter sets of all components in the mixture and all binary interaction parameters of each combination of unlike molecules. For associating molecules a pure component parameter set of five parameters (segment number m_i_^seg^, segment diameter σ_i_, dispersion energy parameter u_i_k_B_^–1^, association energy ε^AiBi^k_B_^–1^, and association volume κ^AiBi^) are required.^13,14^ These pure-component parameters for the APIs have been derived in this work via our ML approach (see Section 2.2).^11^ Moreover, calculation for mixtures requires a binary parameter *k*_ij_ describing the interactions between each pair of molecules i and j. These binary parameters have also been derived from our ML approach (see Section 2.2) or fitted to one solubility data point. Based on the so-obtained pure-component parameter sets and binary parameters, PC-SAFT was used to calculate the API activity coefficients in eq 1 for any solvent or solvent mixtures and thus the API solubility in these solvents. More details about the theoretical background are given in the literature.^9,15,16^

Activity coefficients are a direct measure for the molecular interaction in a liquid. As API polymorphs only differ in their solid phase, they have the same activity coefficients and therewith also the same PC-SAFT parameters (both pure-component parameters and binary parameters). Polymorphs only differ in their melting properties. Thus, once the PC-SAFT parameters are known, the solubilities of different polymorphs are directly accessible by eq 1 just using the melting properties of the target polymorph.^17,18^

The melting properties of the API (*T*_API_^SL^, Δ*h*_API_^SL^, Δ*c*_p_^SL^) were taken from the literature. If Δ*c*_p_^SL^ has not been measured in the respective literature source it was approximated by Δ*s*_API_^SL^ = Δ*h*_API_^SL^/*T*_API_^SL^, which is a common approximation in the literature.^19^

### 2.2 ML Framework

For the prediction of PC-SAFT parameters, the ML framework described in^12^ was modified to be applicable to API-solvent systems. The pure-component parameter sets of the API are ML-derived using the first level Neural Network (NN) ensemble. Pure-component parameter sets of the solvents were taken from literature or in-house databases.

The second level NN-ensemble was then used to access all binary interaction parameters needed to obtain a full parametrization of the system (one in case of API solubility in pure solvents, two in case of API solubility in binary solvent mixtures). In case of binary solvent mixtures, the binary interaction parameter between the solvents was taken from literature/in-house databases. It is worth mentioning that the binary interaction parameter between the two solvents could also be determined using the second level NN-ensemble if no literature parameters are available. The adapted ML framework used in this work based on the ML framework trained in our previous work is visualized in Figure 1.^11^ Having the full parametrization of the respective system at hand, the API solubility can be calculated according to eq 1, under the knowledge of the melting properties of the API (experimentally determined). The key details of the ML framework are given in the Supporting Information.

**Figure 1:**
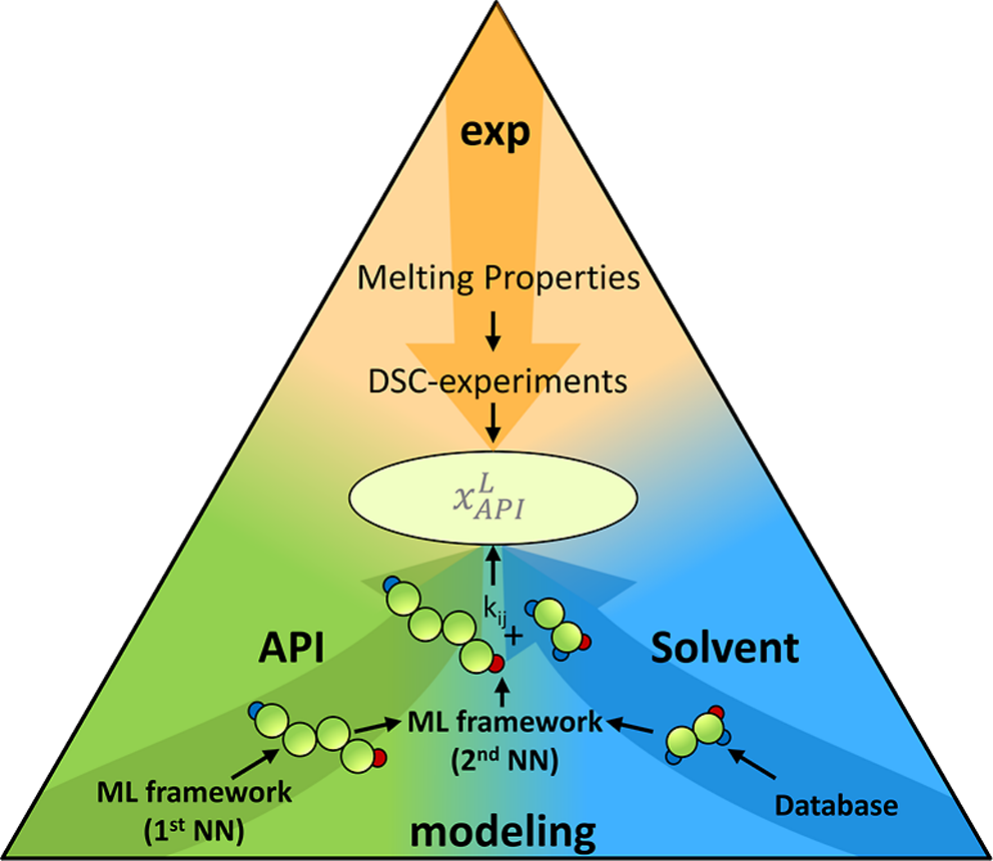
Schematic workflow for the application of the ML framework (developed in our previous work) to predict PC-SAFT parameters, which are used to calculate API solubility. Based on the molecular structure of the API, the PC-SAFT pure-component parameter set can be obtained using the NN-ensemble of the first level. In combination with PC-SAFT pure-component parameter set for the solvent taken from literature and the melting properties of the API (obtained by e.g. DSC experiments), the API solubility can be calculated.

## 3 PC-SAFT Parameters and Melting Properties

Table 1 lists the ML-derived PC-SAFT pure-component parameter sets of the APIs (as described in Section 2.2). As the developed ML approach has been trained only on 2B association, all ML-predicted pure-component parameter sets are either nonassociating or following the 2B association scheme. Electrolyte solutions (e.g., FeSSIS or FaSSIF) cannot be predicted with this approach as additional ePC-SAFT (electrolyte PC-SAFT) parameters would be required for both, the API and the solvent, as well as any other electrolytes in solution (e.g., buffer salts).^20^

**Table 1:** ML-Derived PC-SAFT Pure-Component Parameters^12^ and Molecular Weights (*M*_w_) for all APIs Used in This Work^a^

API	ID	m_i_^seg^	σ_i_/Å	u_i_k_B_^–1^/K	ε^AiBi^ k_B_^–1^/K	κ^AiBi^	assoc. scheme
aceclofenac	ACF	8.68	3.21	317.5	2649.8	0.046	2B
aspirin	ASP	8.12	2.81	258.7	1825.5	0.008	2B
atenolol	ATN	9.22	2.85	316	2977.7	0.018	2B
benorilate	BEN	8.86	3.1	283.4	2661.8	0.003	2B
bifendate	BIF	9.31	3.39	294.9	0	0.01	2B
bifonazole	BFZ	7.57	3.56	319.7	0	0	-
cimetidine	CIM	7.74	3.24	352.4	2033.4	0.013	2B
cinnerazine	CIN	12.01	3.33	247.9	905.6	0.017	2B
dapsone	DAP	6.51	3.05	323.6	1992.6	0.023	2B
dipyridamole	DPA	10.76	3.33	298.62	1802.9	0.007	2B
estrone	EST	6.76	3.69	334.4	2270.1	0.014	2B
fenofibrate	FFB	9.26	3.53	275.9	0	0.02	2B
ibuprofen	IBU	7.28	3.33	295.4	2300.2	0.045	2B
mefenamic acid	MAC	8.98	2.89	271.8	2151	0.005	2B
nadolol	NDO	6.86	3.85	327.8	2225.8	0.009	2B
nimesulide	NIM	6.82	3.49	351.8	1094.8	0.011	2B
paracetamol	PCM	6.46	2.89	278.6	4338.2	0.009	2B
rivaroxaban	RIV	9.53	3.05	348.9	1600.4	0.01	2B
ropivacaine	RVC	6.00	3.88	313.4	1446.7	0.005	2B
tetramethylpyrazine	TMP	3.57	3.57	279.8	0	0	-
γ-indomethacin	g-IND	9.52	3.13	305.0	1019.6	0.033	2B

a Melting properties were taken from the literature (see Table 2). The corresponding PC-SAFT pure-component parameters of the solvents are given in the Supporting Information.

**Table 2:** Melting Properties and Molecular Weights of the APIs Considered in This Work. Δ*c*_p_^SL^ Values with Stars Were Approximated Using Δ*s*_API_^SL^

API	ID	Lit	*T*_API_^SL^/K	Δ*h*_API_^SL^/(kJ mol^–1^)	Δ*c*_p_^SL^/(J mol^–1^K^–1^)	*M*_w_/(g mol^–1^)
aceclofenac	ACF	^21^	426.0	49.3	115.7*	354.2
aspirin	ASP	^22^	409.0	32.6	79.6*	180.2
atenolol	ATN	^23^	424.5	36.6	86.2*	266.3
benorilate	BEN	^24^	451.9	44.5	98.5*	313.3
bifendate	BIF	^25^	434.5	63.8	146.8*	418.4
bifonazole	BFZ	^23^	423.0	37.5	88.7*	310.4
cimetidine	CIM	^26^	414.2	35.5	85.7*	252.3
cinnarizine	CIN	^27^	394.0	37.1	113.6	368.5
dapsone	DAP	^28^	450.4	21.4	47.5*	248.3
dipyridamole	DPA	^29^	412.5	37.9	91.9*	504.6
estrone	EST	^23^	527.1	45.1	85.6*	270.4
fenofibrate	FFB	^30^	354.0	33.5	124.3	360.8
ibuprofen	IBU	^31^	350.2	25.5	50.3	206.3
mefenamic acid	MAC	^23^	503.1	71.2	141.5*	241.3
nadolol	NDO	^23^	403.4	53.0	131.4*	309.4
nimesulide	NIM	^23^	422.2	47.4	112.3*	308.3
paracetamol	PCM	^32^	441.6	28.6	99.8	151.2
rivaroxaban	RIV	^33^	505.6	50.2	99.3*	435.9
ropivacaine	RVC	^34^	420.8	30.1	71.6*	274.4
tetramethylpyrazine	TMP	^35^	358.6	16.4	45.8*	136.2
γ-indomethacin	g-IND	^36^	433.4	39.3	117.0	357.8

### 3.1 Definitions of Statistical Evaluation Metrics

Deviations between two values (*A*_i_ and *B*_i_) of the same property (e.g., solubility, density, mole fractions of vapor phase) but obtained by two different methods (e.g., from ML-calculations, PC-SAFT calculations, or experimental data) were evaluated by means of absolute average relative deviation (AARD), and mean absolute error (MAE) according to eqs 3 and 4).

3 $$AARD=\frac{1}{n_{0}}\sum_{i=1}^{n_{0}}\left|1-\frac{A_{i}}{B_{i}}\right|\cdot 100$$

4 $$MAE=\frac{\sum_{i=1}^{n_{0}}\left|A_{i}-B_{i}\right|}{n_{0}}$$

## 4 Results and Discussion

### 4.1 API Solubility in Single Solvents

API solubility calculations at various temperatures were performed (described in Section 2.1) based on API pure-component parameter sets derived from the ML framework (see Figure 1) in combination with solvent pure-component parameter sets and melting properties of the API taken from literature/in-house databases. In total, the solubility of 21 different APIs has been calculated using the ML-derived API PC-SAFT parameters and compared to a total of 641 solubility data points covering a wide temperature range and a great variety of solvents. Two different scenarios were considered to prove the flexible use of the ML framework used for the calculation of API solubility:

1. The API solubility in different solvents was calculated according to eq 1 using ML-derived pure-component parameters of the API (first level of the ML framework, see Figure 1), known pure-component parameters of the solvent, ML-derived binary interaction parameters (second level of the ML framework, see Figure 1), and experimental melting properties taken from the literature.
2. The API solubility in different solvents was calculated as in scenario 1 but the binary interaction parameter between API and solvent was fitted to one experimental solubility data point.

Scenario 1 shows a solubility prediction using only the melting properties of the API, which can be obtained, e.g. by simple DSC measurements.

Scenario 2 requires one solubility data point. However, compared to conventional fitting methods that need at least six solubility data points at various temperatures to determine PC-SAFT parameters, it significantly reduces the experimental effort. This scenario also examines how sensitive the solubility calculations are to including solubility data. The binary interaction parameters obtained for the two scenarios are given in the Supporting Information.

The results of the solubility calculations for both scenarios are compared to experimental solubility data from literature using parity plots (see Figure 2A for scenario 1 and Figure 2B for scenario 2). The results clearly show, that including an experimental solubility data point improves the reliability in the calculations significantly (increase of *R* ^2^ from 0.83 to 0.98 for the whole data set, see Table 3). This improvement can be explained by the adjustment of the binary interaction parameter from ML-predicted to a value fitted to an experimental data point at one temperature. This dramatically improves modeling accuracy.

**Figure 2:**
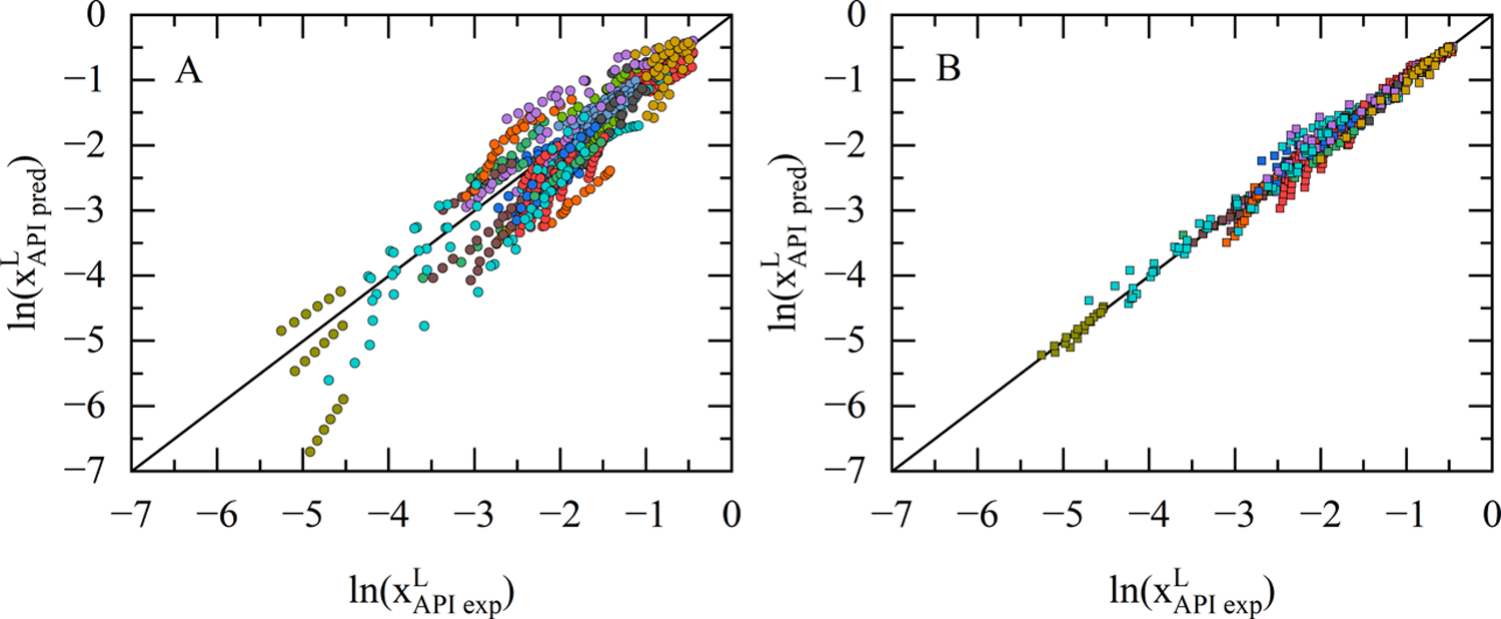
Parity plots for the calculation of 641 solubilities of 21 different APIs in various solvents at different temperatures. Values are given by natural logarithm of the solubility measured in mole fraction. Experimental solubilities were taken from literature^21–37^ (details are given in the Supporting Information). The left diagram (A) shows the calculations using just the chemical structure of the respective API and the melting properties (scenario 1), whereas the right diagram (B) shows calculations based on one solubility of each API at one temperature per binary system (scenario 2).

**Table 3:** Statistical Metrics for Solubility Calculated in the Different Scenarios^a^

*x* ^L^_API_	scenario 1	scenario 2
*R* ^2^	0.83	0.98
MAE	0.345	0.077
AARD/%	18.12	4.17

a The calculations were performed using ML-derived PC-SAFT parameters.

The solubility calculations for ibuprofen, bifendate, fenofibrate, ropivacaine, aspirin, and γ-indomethacin are visualized in more detail in Figure 3. The results reveal that, although the modeling accuracy of scenario 1 (dashed lines in Figure 3) is lower when compared to scenario 2 (solid lines in Figure 3), the individual temperature dependency of solubility can be calculated correctly for most APIs.

**Figure 3:**
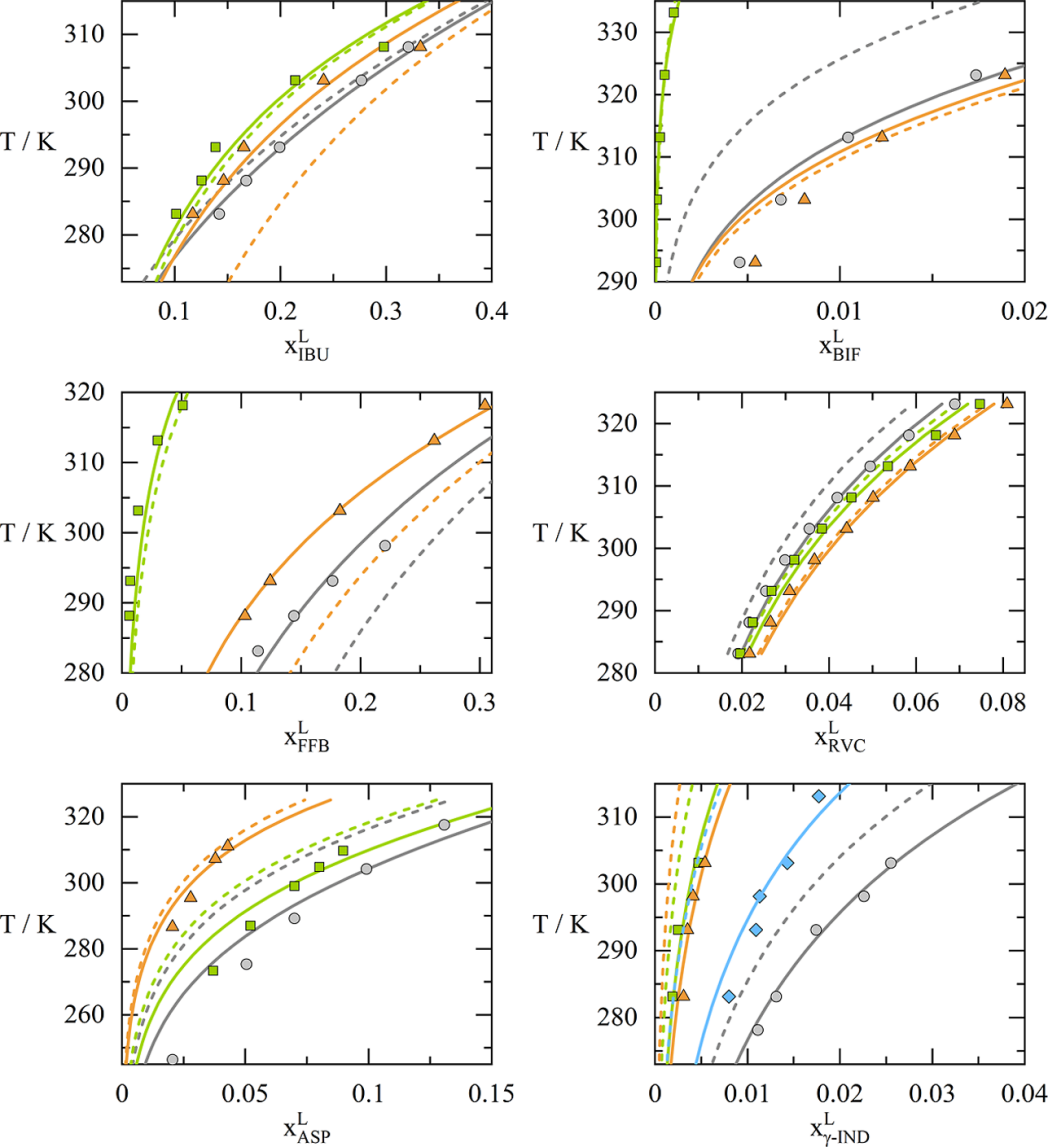
Calculated solubilities of six different APIs (mole fraction) using scenario 1 (dashed lines) and scenario 2 (solid lines). Experimental data (symbols) have been taken from literature^25,30,34,35,37,38^ (details are given in the Supporting Information). IBU: gray-acetone, green-methanol, orange-ethanol; BIF: gray-acetone, green-ethanol, orange-acetonitrile; FFB: gray-acetone, green-ethanol, orange-ethyl acetate; RVC: gray-1-propanol, green-isobutanol, orange-1-pentanol; ASP: gray-acetone, green-2-butanone, orange-isopropyl acetate; γ-IND: gray-acetone, green-2-propanol, orange-ethanol, blue-ethyl acetate.

The results indicate that the solubility of an API in a single solvent can be predicted with an accuracy that is comparable to recent approaches from the literature (*R* ^2^ ∼0.82 in,^4^ *R* ^2^ = 0.9 in,^6^ *R* ^2^ ∼0.87 in^7^) by just using the melting properties of the API and its molecular structure. The true benefits of the new approach are revealed by the results regarding scenario 2 as the accuracy of the calculations has been massively improved (*R* ^2^ increased from 0.83 to 0.98) by adding one single solubility data point per binary system. This data point can be e.g. the aqueous solubility of the API at any temperature, which is anyhow required by the regulatory bodies.

Regarding classical PC-SAFT modeling for API molecules, the results of scenario 2 show a clear improvement as only one solubility data point (instead of at least six) is now sufficient to define all PC-SAFT parameters for a given API-solvent system. Obtaining PC-SAFT parameters of API molecules using the proposed method is thus more efficient and less material consuming than established methods, which can be a crucial factor for the development of pharmaceutical products.

### 4.2 API Solubility in Solvent Mixtures

To show the applicability of our approach also for multicomponent systems, API solubility in solvent mixtures was predicted for four out of the 21 APIs initially considered (namely IBU, PCM, DPA, ASP). Calculations were performed using ML-derived PC-SAFT pure-component parameter sets of the API alongside with existing PC-SAFT pure-component parameter sets of the solvents, and experimental melting properties of the API. The binary interaction parameters for the two API-solvent pairs were either fitted to binary solubility data (if available, see red data points in Figures 4 and 5) or ML-derived (as described in Section 2.2).

**Figure 4:**
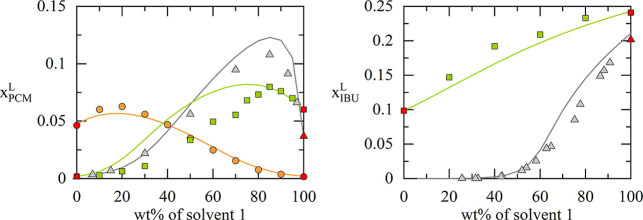
Calculated (lines) and experimental (symbols) solubilities of PCM^39^ and IBU^40,41^ in different solvent mixtures. The *x*-axis shows the weight percentage of the first-named solvent in the respective (API-free) binary solvent mixtures. The red symbols show the API solubilities in the single solvents, which have been used to fit the respective binary interaction parameter between the API and that solvent. For PCM: gray: acetone–water; green: ethanol–water; orange: water-2-propanol. For IBU: gray: ethanol–water; green: ethanol-1,2-propanediol. All data was measured and modeled at 298.15 K.

**Figure 5:**
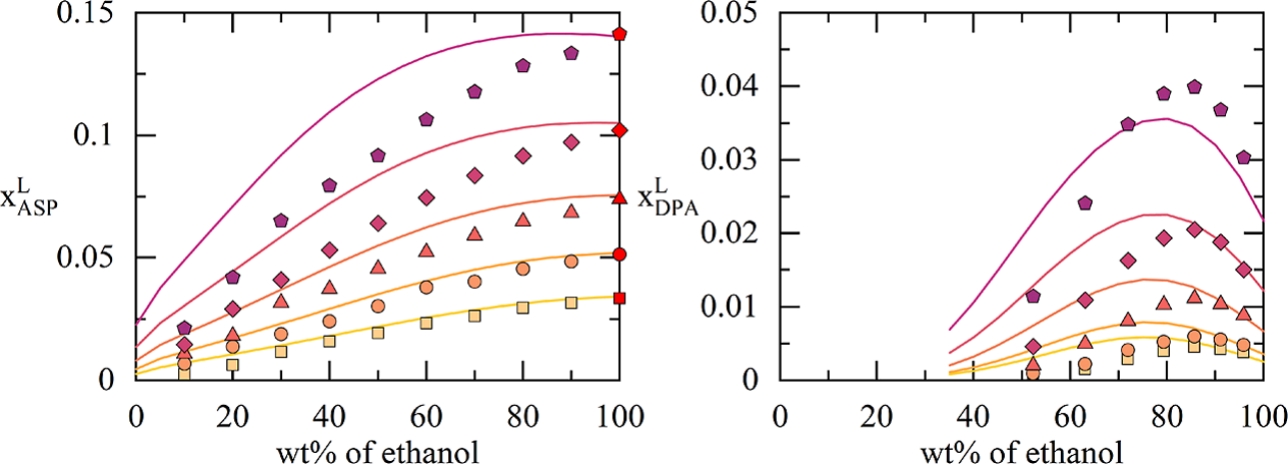
Calculated (lines) and experimental (symbols) solubilities of ASP^42^ and DPA^43^ in one solvent mixture at different temperatures. Solvent mixture for ASP: ethanol-methylcyclohexane; Solvent mixture for DPA: ethanol–water. The *x*-axis shows the weight percentage of ethanol in the (API-free) binary solvent mixture. The red symbols show the API solubilities in the single solvents, which have been used to fit the respective binary interaction parameter between the API and that solvent. Temperatures for ASP (from bottom to top): 283.15 K, 293.15 K, 303.15 K, 313.15 K, 323.15 K. Temperatures for DPA (from bottom to top): 293.15 K, 298.15K, 308.15 K, 318.15 K, 328.15 K.

Aside from the two binary interaction parameters for the API-solvent pairs, one additional binary interaction parameter characterizing the interaction between the two solvents is required, which was fitted to vapor–liquid-equilibrium data of the respective binary solvent system (if available) or ML-derived via the second level of our ML framework.

Having obtained all PC-SAFT parameters of the API-solvent 1-solvent 2 systems, two main aspects were analyzed: the transferability of the ML-derived API parameters to different solvents and solvent systems, and the accuracy of the temperature dependency of solubility.

In Figure 4, solubility calculations of IBU and PCM in different solvent mixtures are shown and compared to experimental data from the literature. The results reveal that the API solubility in the different solvent mixtures can be predicted in good agreement with the experimental data using the ML-derived parameters. The solubility maxima of PCM are described in almost quantitative agreement. Further, the temperature dependency of the solubility of ASP and DPA was predicted (results illustrated in Figure 5) over a temperature range of 293.15 to 328.15 K (DPA) and 283.15 to 323.15 K (ASP) revealing a good agreement with experimental data for these ternary systems. These examples show the ability of the ML framework to predict complex ternary systems using minimal experimental inputs (API solubility in the single solvents at one temperature as well as the melting properties of the API). It is remarkable, that the calculations even correctly reproduce the nonmonotonous behavior in some solvent mixtures, which show solubility maxima or turning points.

The calculations performed in this section show the straightforward application of the ML framework in different scenarios of data availability. From a process-development point of view, the ML framework provides PC-SAFT parameters at the very beginning of process development (solvent screening) as well as in later steps demanding more complex (multicomponent) calculations. These calculations can then be executed guided by key solubility measurements based on the initial screening. Using this approach, which combines predictive thermodynamic calculations and key experiments, solubility synergies in solvent mixtures can be efficiently detected. This allows for an accelerated development of pharmaceutical formulations as the solubility of an API can be tuned by adjusting the solvent(s).

## 5 Conclusion

This work applied ML-derived PC-SAFT parameters for APIs to predict their solubility in single solvents and solvent mixtures. Using the PC-SAFT parameters of the solvents, which are available through various databases, these calculations only require the molecular structure and melting properties of the API. Without using any experimental solubility data, this approach already achieves a reasonable accuracy of predicted solubilities (*R* ^2^ = 0.83) using ML-derived pure-component parameters of the API as well as ML-derived binary interaction parameters between APIs and solvents. Introducing just a single experimental solubility data point per binary system significantly improves the quality of the calculations (*R* ^2^ = 0.98) by using this data point to regress the binary interaction parameter instead of using the ML framework.

This work proved that a combination of ML-predicted PC-SAFT parameters and only one experimental solubility data point ensures high reliability for solubility predictions in early development stages. The method remarkably reduces the experimental burden compared to traditional PC-SAFT modeling, which so far required several experimental data points for parameter fitting of both, pure-component parameters as well as binary interaction parameters.

Using the same PC-SAFT pure-component parameters but different melting properties of different polymorphs, the proposed approach even enables predicting the solubility of different API polymorphs. This is not possible using other ML approaches, which directly predict the solubility from the molecular structure of an API. Besides API solubility, also other thermodynamic properties such as liquid–liquid separation or solvent sorption can be accessed using the predicted pure-component parameters obtained with the ML framework. This allows for a more thorough analysis of the API than just replacing solubility measurements. In addition, the approach proves to be a powerful tool not only for single-solvent systems but also for more complex solvent mixtures offering accurate solubility calculations in mixed solvents without the need for further experimental data.

## Supplementary Material



## Notes

Alongside this article Supporting Information is published. It is covering the PC-SAFT pure-component parameter sets of all solvents used including their literature sources, as well as all PC-SAFT binary interaction parameters calculated in the two scenarios defined in this (including the literature sources of the experimental solubility data). The last section of the Supporting Information contain some key insights into the setup of the ML framework used for predicting the PC-SAFT parameters (PDF)
