# Supplementary material for: Combining PC-SAFT and ML to Access Unknown API Solubilities
Source: Mol Pharm. 2026 Apr 20;23(5):3062–9. doi: 10.1021/acs.molpharmaceut.5c01889 (PMC13147324; doi:10.1021/acs.molpharmaceut.5c01889)
Supplement: Supplementary file 1 [file mp5c01889_si_001.pdf]

Combining PC-SAFT and ML to access unknown API solubilities

Jonas Habicht[a], Gabriele Sadowski[a,b,*], and Christoph Brandenbusch[a,*]

Supporting information

[a]*TU Dortmund University, Laboratory of Thermodynamics, Department of Biochemical and Chemical Engineering, Emil-Figge-Str. 70, 44227 Dortmund, Germany*

[b]*amofor GmbH, Otto-Hahn-Strasse 15, 44227 Dortmund, Germany*

[*]*corresponding authors:*

gabriele.sadowski@tu-dortmund.de

christoph.brandenbusch@tu-dortmund.de

Table 1: PC-SAFT pure-component parameter sets and molecular weights for the solvents used to calculated the API solubilities.

Solvent	m_i^{seg}	σ_i / Å	$u_i k_B^{-1}$ / K	$\varepsilon^{AiBi} k_B^{-1}$ / K	κ^{AiBi}	assoc. scheme	M_w / $gmol^{-1}$
1,2-propanediol	3.97	2.99	241.4	1467.0	0.1030	4B	76.05
1-butanol	4.21	3.07	219.9	1890.7	0.0277	2B	74.12
1-octanol	4.36	3.72	262.7	2754.8	0.0022	2B	130.23
1-pentanol	3.63	3.45	247.3	2252.1	0.0103	2B	88.15
1-propanol	3	3.25	233.4	2276.8	0.0153	2B	60.10
2-butanol	3.32	3.34	227	2255.9	0.0110	2B	74.12
2-butanone	3.07	3.39	249.8	0.0	0.0100	2B	72.11
2-propanol	3.09	3.21	208.4	2253.9	0.0247	2B	60.10
acetone	2.89	3.23	247.4	0.0	0.0100	2B	58.08
acetonitrile	2.33	3.19	311.3	0.0	0.0100	2B	41.05
butyl acetate	4.04	3.52	240.9	0.0	0.0000	none	116.16
ethanol	2.38	3.18	198.2	2653.4	0.0324	2B	46.07
ethyl acetate	3.54	3.31	230.8	0.0	0.0100	2B	88.11
ethyl propanoate	3.84	3.4	232.8	0.0	0.0000	none	102.13
heptane	3.5	3.8	237.6	0.0	0.0000	none	100.2
isobutanol	3.26	3.38	238.6	2370.7	0.0068	2B	74.07
isopentanol	3.18	3.62	258.5	2593.3	0.0038	2B	88.09
isopropyl acetate	3.61	3.48	231.9	0.0	0.0100	2B	102.13
methanol	2.23	2.8	166.9	2668.1	0.0896	2B	32.04
methyl acetate	3.33	3.13	228.2	0.0	0.0100	2B	74.08
methyl isobutyl ketone	3.36	3.68	259.9	0.0	0.0100	2B	100.16
pentyl acetate	4.31	3.6	243.9	0.0	0.0100	2B	130.19
propyl acetate	3.96	3.38	229.7	0.0	0.0100	2B	102.13
water*	1.2	2.79	354	2425.7	0.0451	2B	18.02

*for water the segment diameter is calculated as a temperature dependent function ($\sigma_i(T) = 2.7927 + 10.11e^{-0.01775T} - 1.417e^{-0.01146T}$)

Table 2: PC-SAFT binary interaction parameters of the API-solvent combinations of the two scenarios in this work.

API	solvent	$kij_{,scen\ 1}$	$kij_{,scen\ 2}$	Lit (exp data)
ASP	acetone	-0.036	-0.0281	[1]
ASP	2-butanone	-0.037	-0.0312	[1]
ASP	isopropylacetate	-0.039	-0.0389	[1]
ASP	ethanol	-0.0644	-0.0943	[1]
ASP	methylisobutylketone	-0.029	-0.03	[1]
ATN	water	-0.016	-0.0277	[2]
ATN	ethanol	-0.0859	-0.0552	[2]
ATN	1-octanol	-0.0336	-0.054	[2]
BEN	methanol	-0.011	-0.021	[3]
BEN	isopropylacetate	-0.033	-0.017	[3]
BEN	acetone	-0.025	-0.017	[3]
BEN	ethylacetate	-0.026	-0.005	[3]
BFZ	water	-0.0414	-0.0236	[2]
BFZ	ethanol	-0.035	-0.0165	[2]
BFZ	1-octanol	-0.019	-0.0129	[2]
BIF	acetone	-0.0312	-0.0187	[4]
BIF	ethanol	-0.0333	-0.0211	[4]
BIF	methanol	-0.0154	-0.0155	[4]
BIF	1-propanol	-0.0094	-0.0191	[4]
BIF	1-butanol	-0.0039	-0.0179	[4]
BIF	1-pentanol	-0.0104	-0.0176	[4]
BIF	2-propanol	-0.0188	-0.0179	[4]
BIF	methyl acetate	-0.0345	-0.0171	[4]
BIF	ethyl acetate	-0.0386	-0.0158	[4]
BIF	propypl acetate	-0.0363	-0.0149	[4]
BIF	pentyl acetate	-0.0323	-0.008	[4]
BIF	acetonitrile	-0.0204	-0.0215	[4]
CIM	ethanol	-0.0523	-0.0443	[5]
DAP	ethanol	-0.0204	-0.0202	[6]
DAP	methanol	-0.0204	-0.007	[6]
DAP	methylacetate	-0.0365	-0.0203	[6]
DAP	ethylacetate	-0.039	-0.0243	[6]
DAP	butylacetate	-0.0321	-0.0182	[6]
DPA	methanol	-0.0088	-0.0069	[7]
DPA	ethanol	-0.0231	-0.0077	[7]
DPA	1-propanol	-0.0067	-0.0068	[7]
DPA	2-propanol	-0.0141	-0.0065	[7]
EST	ethanol	0.003	-0.003	[2]
EST	1-octanol	-0.0089	-0.0093	[2]
FFB	acetone	0.0018	-0.0149	[8]
FFB	acetonitrile	0.0149	-0.0235	[8]
FFB	methanol	0.0139	0.0056	[8]
FFB	ethanol	-0.0041	-0.0068	[8]

FFB	1-propanol	0.0043	-0.0141	[8]
FFB	2-propanol	0.0109	-0.0105	[8]
FFB	ethylacetate	0.0031	-0.0104	[8]
g-IND	acetone	-0.0254	-0.022	[9]
g-IND	2-propanol	-0.0306	-0.0242	[9]
g-IND	ethanol	-0.0454	-0.0313	[9]
g-IND	ethylacetate	-0.0345	-0.0216	[9]
IBU	acetone	-0.0135	-0.0117	[10]
IBU	methanol	-0.0079	-0.0099	[10]
IBU	ethanol	-0.0267	-0.0435	[10]
IBU	ethylacetate	-0.0246	-0.0132	[10]
IBU	2-propanol	-0.0188	-0.036	[10]
IBU	2-butanone	-0.0258	-0.016	[1]
IBU	heptane	-0.0465	0.016	[1]
IBU	12-propanediol	0.007	-0.023	[10]
MAC	ethanol	-0.0978	-0.0712	[2]
NAP	2-propanol	-0.0376	-0.044	[9]
NAP	ethanol	-0.0515	-0.0486	[9]
NAP	methanol	-0.0318	-0.0162	[9]
NDO	ethanol	0.0029	0.0135	[2]
NDO	1-octanol	0.0104	-0.002	[2]
NIM	water	-0.0287	-0.0183	[2]
NIM	ethanol	-0.0169	-0.023	[2]
NIM	1-octanol	-0.0003	-0.018	[2]
RIV	ethanol	-0.0531	-0.0133	[11]
RIV	1-propanol	-0.0186	-0.0123	[11]
RIV	1-butanol	-0.0061	-0.0142	[11]
RVC	methanol	0.0271	0.0547	[12]
RVC	ethanol	0.0066	0.0147	[12]
RVC	1-propanol	0.0088	0.0109	[12]
RVC	2-propanol	0.0185	0.0117	[12]
RVC	1-butanol	0.0223	0.0166	[12]
RVC	isobutanol	0.0045	0.0052	[12]
RVC	2-butanol	0.0095	0.0057	[12]
RVC	1-pentanol	0.002	0.0024	[12]
RVC	Isopentanol	-0.0012	-0.0012	[12]
RVC	ethylformate	0.027	0.0057	[12]
RVC	ethylacetate	0.031	0.0022	[12]
RVC	acetonitrile	0.034	0.0068	[12]
TMP	methanol	-0.035	0.007	[13]
TMP	ethanol	-0.0142	-0.0124	[13]
TMP	1-propanol	-0.012	-0.0029	[13]
TMP	2-propanol	0.0035	-0.0063	[13]
TMP	1-butanol	0.0019	0.0114	[13]

Key details of the ML framework

This section summarizes the key details of the ML strategy relevant for this work, which have been developed in our previous works [14, 15]. Based on the general adjustment of the ML framework (see Figure 1 in the manuscript), two trained neural network ensembles have been applied to predict PC-SAFT pure-component parameter sets (of APIs) (1st NN), and binary interaction parameters of API-solvent combinations (or solvent-solvent combinations) (2nd NN).

1st NN: Prediction of PC-SAFT pure-component parameter sets

A training and validation dataset of 1,033 molecules was considered including associating molecules. All PC-SAFT pure-component parameter sets were already reported in literature. Independently of the molecular structure, associating components were described using one donor association site and one acceptor association site (2B association scheme) to reduce the possible PC-SAFT pure-component parameter sets. If already published in literature, also molecules, which are sometimes modeled with different association schemes (e.g. acetic acid), their 2B parametrization was added for the neural network (NN)-training. Thus, as output of the NN-ensemble, the five PC-SAFT pure-component parameters are calculated always assuming 2B-association if an associating species is present. [15]

As input features, the column entries of the Extended-connectivity fingerprints (ECFPs, initial bit length = 2^{14}) have been used alongside the number of different atoms, the molecular weight, the number of ring structures, and the number of rotatable bonds. Rotatable bonds were defined as single bonds, which are not part of a ring structure and are connected to a non-terminal, non-hydrogen atom. Having removed the redundant zero-columns of the ECFPs in the training set, Standardscaler and Minmaxscaler (using *scikit-learn*, [16]) were applied to perform data preprocessing. Due to the column removal, the input size reduced from $>2^{14}$ to ~3,000 input neurons depending on the train/test-split. [14]

The general NN-structure for the training was designed according to Table 3. To avoid overfitting, dropout layers with a dropout ratio of 0.05-0.4 were added and a weight decay factor was introduced. The training of the neural network ensembles was performed using five-fold cross validation.

Table 3: Neural network-architecture for the neural networks trained to predict PC-SAFT pure-component parameter sets. [15]

Neural Network Parameter	
Number of hidden layers	9
Hidden layer size 1	2048
Hidden layer size 2	1024
Hidden layer size 3-5	512
Hidden layer size 6-7	256
Hidden layer size 8	128
Hidden layer size 9	32
Activation function	SELU[17]
Learning rate	$3.5 \cdot 10^{-5}$
Weight decay	$2.8 \cdot 10^{-5}$
Loss function	Huber Loss[18]
Optimizer	Adam[19]

2nd NN: Prediction of PC-SAFT pure-component parameter sets

To predict the PC-SAFT binary interaction parameters, a neural network ensemble was trained using the PC-SAFT pure-component parameter sets of both molecules and their molecular weights as input. For the 2nd NN all association schemes have been included in the training data (not only 2B) as well as the dipole moment μ_i leading to 20 input features (two times six PC-SAFT pure-component parameters (m_i^{seg}, σ_i, $u_i k_B^{1}$, $\varepsilon^{AiBi} k_B^{-1}$, κ^{AiBi}, and μ_i), two times three numbers of association sites N_A, N_B, N_{AB}, two molecular weights).[15] As the prediction has a lower dimensionality compared to the ECFP-input in the 1st NN, smaller neural networks have been used to predict the binary interaction parameter. The same methods to avoid overfitting, to perform data scaling, and to perform cross validation have been used compared to the 1st NN. The dataset used for NN-training contained 7,300 k_{ij} values and the respective 1,108 pure-component parameter sets of the corresponding molecules for 6,301 binary systems (no API systems). The key details of the NN-architecture of the 2nd NN are given in Table 4.

Table 4: Neural network-architecture for the neural networks trained to predict PC-SAFT binary interaction parameters [15].

Neural Network Parameter	
Number of hidden layers	5
Hidden layer size 1	32
Hidden layer size 2-3	128
Hidden layer size 4	32
Hidden layer size 5	8
Activation function	SELU[17]
Learning rate	$6.5 \cdot 10^{-4}$
Weight decay	$1.5 \cdot 10^{-7}$
Optimizer	Adam[19]

1 References

[1] Hahnenkamp, I.; Graubner, G.; Gmehling, J. Measurement and prediction of solubilities of active pharmaceutical ingredients. *International journal of pharmaceutics*, **2010**, *388,* 73–81.

[2] Domańska, U.; Pobudkowska, A.; Pelczarska, A.; Winiarska-Tusznio, M.; Gierycz, P. Solubility and pKa of select pharmaceuticals in water, ethanol, and 1-octanol. *The Journal of Chemical Thermodynamics*, **2010**, *42,* 1465–1472.

[3] Hu, W.; Shang, Z.; Wei, N.; Hou, B.; Gong, J.; Wang, Y. Solubility of benorilate in twelve monosolvents: Determination, correlation and COSMO-RS analysis. *The Journal of Chemical Thermodynamics*, **2021**, *152,* 106272.

[4] Cui, C.; Wu, H.; Sadowski, G.; Ji, Y. Solubility Measurement and Thermodynamic Modeling of Bifendate in 13 Pure Solvents at Temperatures from 293.15 to 333.15 K. *J. Chem. Eng. Data*, **2024**, *69,* 4445–4453.

[5] Matsuda, H.; Mori, K.; Tomioka, M.; Kariyasu, N.; Fukami, T.; Kurihara, K.; Tochigi, K.; Tomono, K. Determination and prediction of solubilities of active pharmaceutical ingredients in selected organic solvents. *Fluid Phase Equilibria*, **2015**, *406,* 116–123.

[6] Li, W.; Ma, Y.; Yang, Y.; Xu, S.; Shi, P.; Wu, S. Solubility measurement, correlation and mixing thermodynamics properties of dapsone in twelve mono solvents. *Journal of Molecular Liquids*, **2019**, *280,* 175–181.

[7] Wang, S.; Chen, Y.; Gong, T.; Dong, W.; Wang, G.; Li, H.; Wu, S. Solid-liquid equilibrium behavior and thermodynamic analysis of dipyridamole in pure and binary solvents from 293.15 K to 328.15 K. *Journal of Molecular Liquids*, **2019**, *275,* 8–17.

[8] Watterson, S.; Hudson, S.; Svärd, M.; Rasmuson, Å.C. Thermodynamics of fenofibrate and solubility in pure organic solvents. *Fluid Phase Equilibria*, **2014**, *367,* 143–150.

[9] Dohrn, S.; Luebbert, C.; Lehmkemper, K.; Kyeremateng, S.O.; Degenhardt, M.; Sadowski, G. Solvent influence on the phase behavior and glass transition of Amorphous Solid Dispersions. *European journal of pharmaceutics and biopharmaceutics : official journal of Arbeitsgemeinschaft fur Pharmazeutische Verfahrenstechnik e.V*, **2021**, *158,* 132–142.

[10] Gracin, S.; Rasmuson, Å.C. Solubility of Phenylacetic Acid, p -Hydroxyphenylacetic Acid, p -Aminophenylacetic Acid, p -Hydroxybenzoic Acid, and Ibuprofen in Pure Solvents. *J. Chem. Eng. Data*, **2002**, *47,* 1379–1383.

[11] Jeong, J.-S.; Ha, E.-S.; Park, H.; Lee, S.-K.; Kim, J.-S.; Kim, M.-S. Measurement and correlation of solubility of rivaroxaban in dichloromethane and primary alcohol binary

solvent mixtures at different temperatures. *Journal of Molecular Liquids*, **2022**, *357,* 119064.

[12] Wang, Y.; Cai, L.; Du, S.; Cheng, Y.; Zhang, P.; Li, Y.; Xue, F.; Gong, J. Solid-liquid equilibrium of ropivacaine in fourteen organic solvents: An experimental and molecular simulation study. *Journal of Molecular Liquids*, **2022**, *349,* 118163.

[13] Zhang, N.; Li, S.; Yang, H.; Li, M.; Yang, Y.; Tang, W. Measurement and Correlation of the Solubility of Tetramethylpyrazine in Nine Monosolvents and Two Binary Solvent Systems. *J. Chem. Eng. Data*, **2019**, *64,* 995–1006.

[14] Habicht, J.; Brandenbusch, C.; Sadowski, G. Predicting PC-SAFT pure-component parameters by machine learning using a molecular fingerprint as key input. *Fluid Phase Equilibria*, **2023**, *565,* 113657.

[15] Habicht, J.; Sadowski, G.; Brandenbusch, C. An Adaptive ML Framework to Predict PC-SAFT Parameters for Mixtures. *Ind. Eng. Chem. Res.*, **2025**, *64,* 22413–22427.

[16] Pedregosa, F.; Varoquaux, G.; Gramfort, A.; Michel, V.; Thirion, B. Scikit-learn: Machine Learning in Python. *J. Mach. Learn. Res.*, **2011,** 2825–2830.

[17] Kılıçarslan, S.; Adem, K.; Çelik, M. An overview of the activation functions used in deep learning algorithms. *Journal of New Results in Science*, **2021**, *10,* 75–88.

[18] Huber, P.J. Robust Estimation of a Location Parameter. *Ann. Math. Statist.*, **1964**, *35,* 73–101.

[19] Kingma, D.P.; Ba, J. Adam: A Method for Stochastic Optimization. *arXiv preprint (1412.6980)*, **2014**.
